# Systematic review and meta-analysis of efficacy and safety of continuous positive airways pressure versus high flow oxygen cannula in acute bronchiolitis

**DOI:** 10.1186/s12887-022-03754-9

**Published:** 2022-12-03

**Authors:** Jefferson Antonio Buendía, John Edwin Feliciano-Alfonso, Mauricio Fernandez Laverde

**Affiliations:** 1 Research group in Pharmacology and Toxicology ”INFARTO”, Department of Pharmacology and Toxicology, University of Antioquia, Medellín, Colombia; 2 Facultad de Medicina, Universidad de Antioquia, Carrera 51D, Medellín, Colombia; 3 Departamento de Medicina Interna, Universidad Nacional de Colombia, Bogota, Colombia; 4 Unidad de Cuidado Intensivo Pediatrico. Hospital Pablo Tobon Uribe. Medellin. Facultad de Medicina, Universidad CES, Carrera 51D, Medellín, Colombia

**Keywords:** Continuous positive airways pressure, High flow oxygen cannula, Children, Bronchiolitis, Severe bronchiolitis, Continuous positive air pressure, High-flow nasal cannula

## Abstract

**Introduction:**

There are a trend towards increasing use of High-Flow Nasal Cannula (HFNC), outside of paediatric intensive care unit. Give this trend is necessary to update the actual evidence and to assess available published literature to determinate the efficacy of HFNC over Continuous Positive Air Pressure (CPAP) as treatment for children with severe bronchiolitis.

**Methods:**

We searched MEDLINE, EMBASE, LILACS, and COCHRANE Central, and gray literature in clinical trials databases (www.clinicaltrials.gov), from inception to June 2022. The inclusion criteria for the literature were randomized clinical trials (RCTs) that included children < 2 years old, with acute moderate or severe bronchiolitis. All study selection and data extractions are performed independently by two reviewers.

**Results:**

The initial searches including 106 records. Only five randomized controlled trial that met the inclusion criteria were included in meta-analysis. The risk of invasive mechanical ventilation was not significantly different in CPAP group and HFNC group [OR: 1.18, 95% CI (0.74, 1.89), I² = 0%] (very low quality). The risk of treatment failure was less significantly in CPAP group than HFNC group [OR: 0.51, 95% CI (0.36, 0.75), I² = 0%] (very low quality).

**Conclusion:**

In conclusion, there was no significant difference between HFNC and CPAP in terms of risk of invasive mechanical ventilation. CPAP reduces de risk of therapeutic failure with a highest risk of non severe adverse events. More trials are needed to confirm theses results.

**Supplementary Information:**

The online version contains supplementary material available at 10.1186/s12887-022-03754-9.

## Introduction

Bronchiolitis is the most common respiratory disease in childhood, with an incidence of 1 in 10 children in the first year; being the first cause of hospitalization in pediatric age around the world. [1, 2]. Around 8% of all paediatric intensive care unit (PICU) admissions annuall are caused by bronchiolitis [3]. However, this numbers are rising over the last decade [4]. At present, there is no effective treatment for bronchiolitis to avoid admission to PICU and possible intubation; and the available treatments only are supportive therapies [5, 6]. In recent years the use of noninvasive ventilation therapies (NIV), such as the nasal Continuous Positive Air Pressure (CPAP), and the High-Flow Nasal Cannula (HFNC), have emerged as alternatives to orotracheal intubation and conventional invasive ventilation in patients with moderate to severe bronchiolitis [7].

HFNC and CPAP, are high flow system and is able to generate a positive end expiratory pressure [5, 6]. HFNC reduce the upper airway dead space and resistance [5]. HFNC is considered a less invasive treatment than CPAP, better tolerated by the patients, and easier to handle [5, 7]. The NIV historically has reduced intubation rates reducing potentially health care costs [8]. Also, our team recently found that HFNC, concerning conventional nasal cannula, was associated with a slight difference in the number of quality-adjusted life-years in favor of HFNC and with a saving of approximately US$72 per patient. These findings, if projected to the population level in Colombia for 5 years, could mean an estimated savings of US$13,166,071 if the HFNC is adopted for the routine management of all patients with moderate acute bronchiolitis [9, 10]. When comparing HFNC vs. CPAP, the results of two recent systematic reviews are controversial. Dafydd et al. in a systematic review and metanalysis of four randomized controlled trial reported no significant difference in treatment failure was found between CPAP and HFNC (OR 1.64, 95%CI 0.96 to 2.79; p = 0.07) g children up to 24 months of age with a diagnosis of bronchiolitis [11]. However, Wang et al., in a recent meta-analyses shows CPAP was associated with less risk of treatment failure with CPAP regarding HFNC (OR 0,55, 95% ci 0,36 to 0,85) in children with acute lower respiratory infections [12]. There are a trend towards increasing use of HFNC outside of PICU, despite a this lack of evidence over CPAP. Give this trend is necessary to update the actual evidence and to assess available published literature to determinate the efficacy of HFNC over CPAP as treatment for children with bronchiolitis. Having this information will allow optimizing the design of clinical practice guidelines by the government and health insurers.

## Methods

### Search strategy

We searched MEDLINE, EMBASE, LILACS, and COCHRANE Central, and gray literature (in clinical trials databases (www.clinicaltrials.gov) and Google / Google Scholar), from inception to June 2022. We performed manual searches of relevant articles referenced in the eligible studies. There were not language limits. The search strategy is detailed in the Supplemental material.

### Inclusion criteria

The inclusion criteria for the literature were randomized clinical trials (RCTs) that included children < 2 years old, with acute moderate or severe bronchiolitis [6]. We considered studies that compared HFNC with CPAP. We excluded articles that did not meet all the previous criteria about population, intervention, comparison, and outcome of interest. In addition, review conference, letter, comment articles, and so forth; nonrandomized controlled trials; animal experimental study. The trial must also report at least one of the outcomes of interest: invasive mechanical ventilation, failure of therapy or length of stay in hospital and mortality. Studies from any acute hospital setting; paediatric ED, wards or intensive care were included. The primary outcome in our was risk of invasive mechanical ventilation. Secundary outcomes were failure of therapy, length of stay in hospital and mortality.

### Study selection and data extraction

All study selection and data extractions are performed independently by two reviewers (JB and JEF). All titles and abstract were screened using the inclusion criteria. Full text were obtained for those that met the inclusion criteria and articles that do not meet this criteria were excluded. Disagreements were resolved by consensus.

### Risk of bias assessment

Two reviewers (JB and JEF) assessed the risk of bias (RoB) of the included studies with the Cochrane RoB tool [13]. Disagreements were resolved by consensus. The risk of publication bias among the studies was planned to be assessed by visual inspection of the funnel plot figure if we obtained more than 10 studies. To evaluate the quality of the included literature, and the GRADE tool (GDT) was used to evaluate the quality of the included outcomes.

### Data synthesis and statistical methods

For dichotomous outcomes (invasive mechanical ventilation, treatment failure), we calculated the odds ratio (OR) and for continuous outcomes (length of stay) the mean difference, with their 95% confidence interval (95%CI). Heterogeneity was assessed using the I^2^ statistics calculated from Cochran’s Q test.Since we recognise that the studies are based on multiple populations, we chose to use the random-effects model for the analysis, regardless of the I^2^ results. All statistical analysis was performed using Review Manager (RevMan 5.4).

## Results

**Fig. 1:**
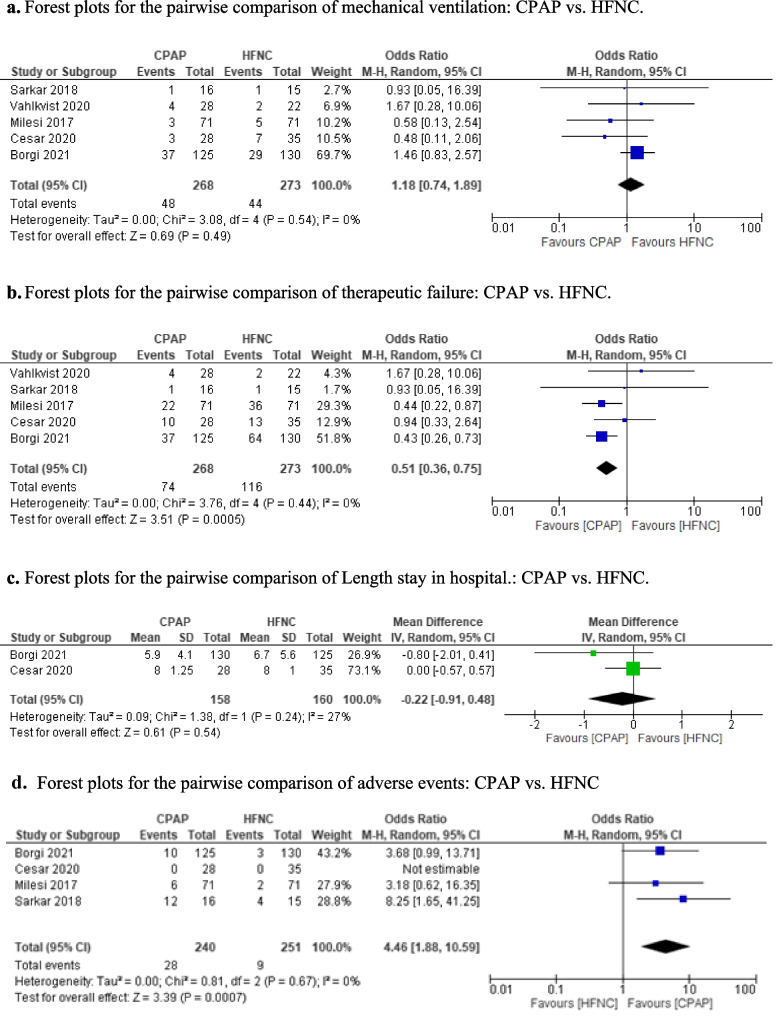
Study flow diagram

The initial searches including 106 records. After deduplication, 75 records were removed. After screening titles and abstracts, 59 records were removed. Therefore, the only five randomized controlled trial that met the inclusion criteria were included in meta-analysis [14–18], see Fig. 1. The list of articles excluded and its reasons is detailed in the supplemental material.

### Characteristics of the included studies

**Fig. 2:**
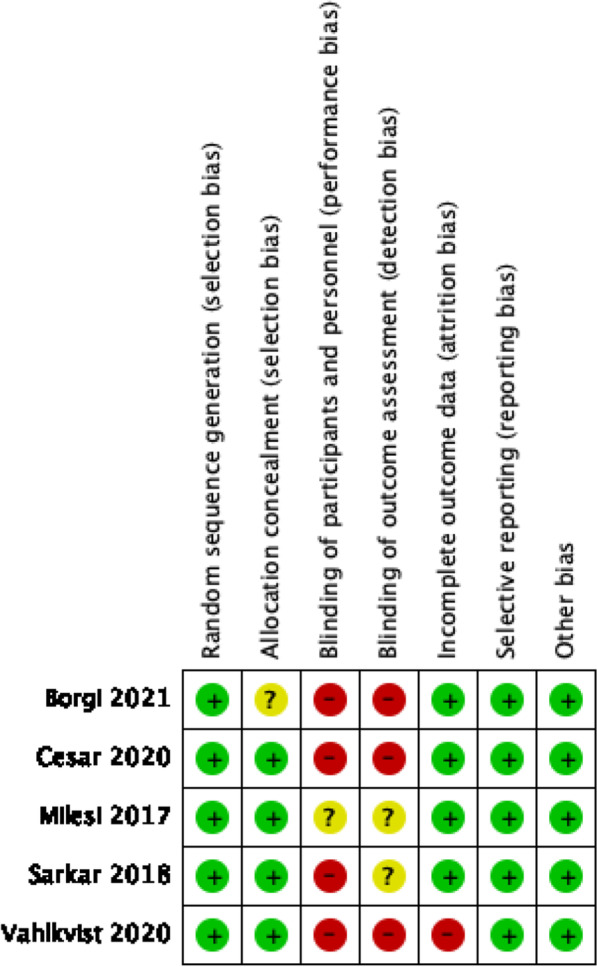
Risk of bias assessment of the included studies

The information of the included studies is in Table 1. The bias of the studies is presented in Table 2. Among the included studies, 2 have a high risk in the integrity of the outcome data. In all of them, it was not possible to mask the treatment given the intervention studied. The studies did not have significant losses of follow-up, Fig. 2. GRADE results were qualified as “very low quality” for invasive mechanical ventilation, mortality, length of stay respectively, Table 2. The number of studies was very low and therefore, the interpretation of its results is limited, and we cannot determine with enough confidence the risk of publication bias.

**Table 1:** Characteristics of included studies

Study	Country	Age	*N*	Setting	Inclusion criteria	Treatment group		Control group	
						**Name**	**Interface**	**Name**	**Interface**
Milési et al. (18)	France	1 d to 6 mon	142	PICU	Bronchiolitis, and moderate to severe respiratory distress	HFNC	Optiflow system	CPAP	Infant Flow Ventilator or FlexiTrunk infant interface
Sarkar et al. (19)	India	28 d to 1 y	31	PICU	Severe bronchiolitis consistent with clinical features, SpO2 < 92% in room air, and RDAI ≥ 11	HFNC	Nasal prong	CPAP	Nasal prong or nasal mask
Vahlkvist et al. (20)	Denmark	< 2 y	50	ED	Bronchiolitis and need for respiratory support	CPAP	Nasal prong	HFNC	Nasal prong
Borgi et al. (21)	Tunisia	7 days to 6 months	255	PICU	Clinical diagnosis of bronchiolitis of moderate severity Wang modified score ≥ 10	CPAP	Nasal mask or nasal prongs	HFNC	Nasal cannula
Cesar et al. (8)	Brazil	< 9 mon	63	PICU	Diagnosis of bronchiolitis of moderate severity or greater	CPAP	Nasal prong	HFNC	Nasal cannula

*CPAP* Continuous positive airway pressure, *EDIN* Neonatal pain and discomfort scale, *HFNC* Humidified high-flow nasal cannula, *mWCAS* modified Wood’s clinical asthma score, *PICU* Pediatric intensive care unit, *SOT* Standard oxygen therapy, *SpO*_*2*_ arterial pulse oximetry, *SPOC* Standard pediatric observation charts, *RDAI* Respiratory distress assessment index

**Table 2:** GRADE summary of findings table with all the outcomes

Outcomes	№ of participants (studies) Follow-up	Certainty of the evidence (GRADE)	Relative effect (95% CI)	Anticipated absolute effects	
				**Risk with HFNC**	**Risk difference with CPAP**
Mechanical Ventilation	541 (5 RCTs)	⨁◯◯◯ Very low^a,b,c^	**OR 1.18** (0.74 to 1.89)	161 per 1000	**24 more per 1000** (37 fewer to 105 more)
Therapeutic failure	541 (5 RCTs)	⨁◯◯◯ Very low^a,c,d,e^	**OR 0.51** (0.36 to 0.75)	425 per 1000	**151 fewer per 1000** (215 fewer to 68 fewer)
Lenght stay in hospital	318 (2 RCTs)	⨁◯◯◯ Very low^a,b,c^	-	The mean lenght stay in hospital ranged from **6.7 to 8** SD	MD **0.22 SD lower** (0.91 lower to 0.48 higher)
Adverse events	519 (4 RCTs)	⨁⨁◯◯ Low^a,c,d^	**OR 4.46** (1.88 to 10.59)	36 per 1000	**106 more per 1000** (29 more to 247 more)

**GRADE Working Group grades of evidence**

**High certainty:** we are very confident that the true effect lies close to that of the estimate of the effect

**Moderate certainty:** we are moderately confident in the effect estimate: the true effect is likely to be close to the estimate of the effect, but there is a possibility that it is substantially different

**Low certainty:** our confidence in the effect estimate is limited: the true effect may be substantially different from the estimate of the effect

**Very low certainty:** we have very little confidence in the effect estimate: the true effect is likely to be substantially different from the estimate of effect

***CI*** Confidence interval, ***MD*** Mean difference, ***OR*** Odds ratio

Explanations

*The risk in the intervention group (and its 95% confidence interval) is based on the assumed risk in the comparison group and the **relative effect** of the intervention (and its 95% CI)

^a^Studies had high or unclear risk of selection and performance bias

^b^Inconsistence. According to the confidence interval there are differences in the direction of effect . CPAP can increase or reduce the risk

^c^Imprecise. Due to low sample size, the 95% CI was very wide

^d^Inconsistence. The studies with the largest sample size in the review (Milesi 2017 and Borgi 2021) show a effect of CPAP, while the other 3 show no differences

^e^Indirectness. In most studies this outcome is has a component of subjectivity because there was an option for individual clinicians to independently decide that participants had failed a particular therapy, in addition to objective markers such as worsening of physiological parameters

### Meta-analysis of outcomes

#### Invasive mechanical ventilation

**Fig. 3:**
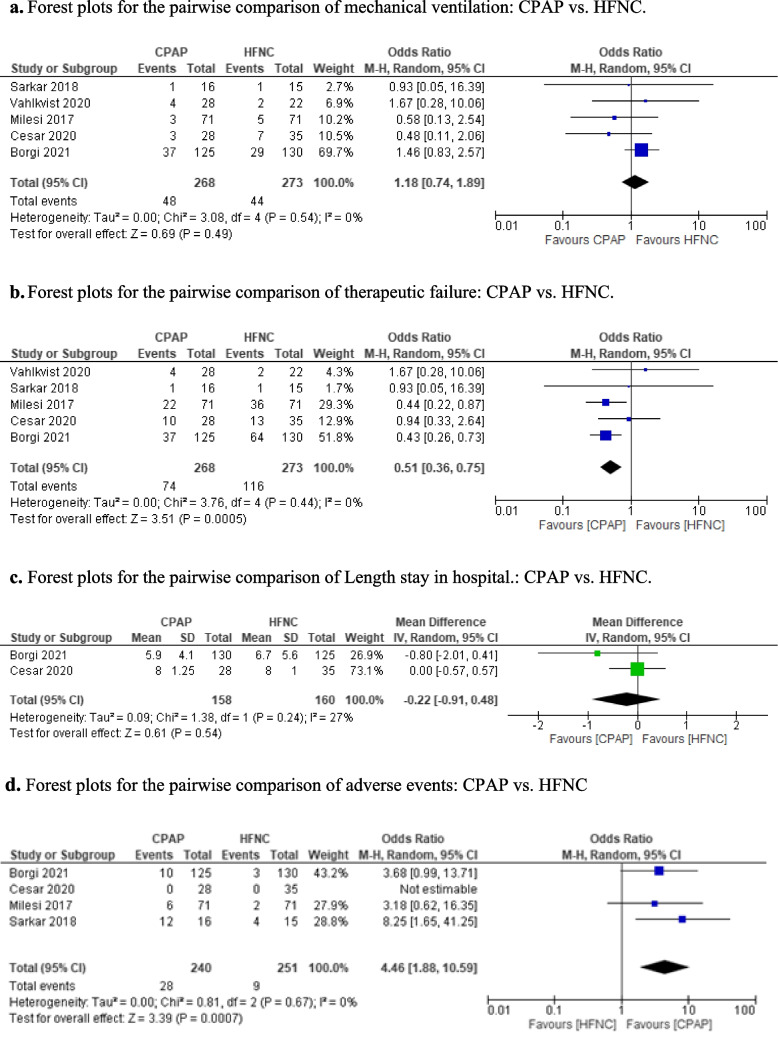
Forest plots for the pairwise comparison of CPAP vs. HFNC. **a**Forest plots for the pairwise comparison of mechanical ventilation: CPAP vs. HFNC. **b** Forest plots for the pairwise comparison of therapeutic failure: CPAP vs. HFNC. **c** Forest plots for the pairwise comparison of Length stay in hospital.: CPAP vs. HFNC. **d** Forest plots for the pairwise comparison of adverse events: CPAP vs. HFNC.

A total of 5 RCTs were included, including 541 children analysis [14–18]. The risk of invasive mechanical ventilation was not significantly different in CPAP group and HFNC group [OR: 1.18, 95% CI (0.74, 1.89), I² = 0%] (very low quality), Fig. 3a.

#### Treatment failure

A total of 5 RCTs were included, including 541 children analysis [14–18]. The risk of treatment failure was less significantly in CPAP group than HFNC group [OR: 0.51, 95% CI (0.36, 0.75), I² = 0%] (very low quality), Fig. 3b.

#### Length of stay

A total of 2 RCTs were included, including 318 children analysis [17, 18]. There are not differences significantly in the length of stay between CPAP group and HFNC group [MD =: -0.22, 95% CI (-0.91, 0.48), I² = 27%] (very low quality), Fig. 3c.

#### Mortality

A total of 1 RCTs were included, including 255 children analysis [17]. There are not differences significantly in the mortality betweem CPAP group and HFNC group [OR: 3.14, 95% CI (0.13, 77.92), I² = NA] (very low quality).

#### Adverse events

A total of 5 RCTs were included, including 541 children analysis [14–18]. The risk of adverse events (skin lesions, poor system tolerance,abdominal distencion) was higher significantly in CPAP group than HFNC group [OR: 3.39, 95% CI (1.48, 7.77), I² = 7%] (low quality), Fig. 3d.

## Discusion

Our review found that there are not differences between CPAP and HFNC in the risk of mechanical ventilation and mortality. CPAP was associated with less risk of treatment failure but with higher adverse events than HFNC. Overall, the certainty of evidence was low because of the small number of trials and variability of methodology among studies.

This results are consistent with recenty evidence of CPAP and HFNC in acute lower respiratoy infection in children. Wang in a systematic review and bayesian network meta-analysis found that, compared with standard oxygen therapy, CPAP was associated with a lower risk of intubation (OR: 0.40, 95% CrI: 0.16–0.90) [12]. But there were no significant differences between these tretments in intubation rate, and in-hospital mortality. In the indirect comparisons,for intubation rate, the SUCRA for BIPAP, CPAP,HFNC, and, standard oxygen therapy (SOT) were 88.1, 73.0, 28.7, and 10.2%, respectively [12].

The treatment failure result has a special consideration in its interpretation. Its definition is not homogeneous among all the studies, in addition to the fact that within it the decision to declare the presence or not of failure is subjective since it does not totally depend on objective ventilatory parameters. As occur in this cases we were limited to the data reported in the included studies. Perhaps the best way to validate a result in this type of outcomes is by observing its correlation with more objective results such as intubation or mortality. In this case, CPAP was not associated with less risk of mortality and mechanical ventilation. It´s possible that this degree of subjectivity in the measurement of treatment failure in the studies biases the result and no be a reliable mesure to assess the effectiveness of theses treatments.

Regarding previous systematic reviews published in children with bronchiolitis, the principal difference is the inclusion of Borgi’s study [11, 19]. This study included 268 participants and is the largest sample size conducted to date, which explains its greater weight in the meta-analysis. In this study, the success of the treatment was significantly higher in the CPAP/NPPV group (70.4% [61.6- 78.2%) comparing to HFNC group (50.7% [41.9- 59.6%]) [17]. However there are not statistically significant differences are reported in mechanical ventilation or adverse events. As was mentioned before is controvertible this differences only in success of treatment and not in other “hard” outcomes.

In patients with severe bronchiolitis HFNC could be an alternative to CPAP, especially in low resources settings, due to absence of significative differences in mechanical ventilation or mortality, with less risk of adverse events. Both alternatives had reduced the risk for intubation, and the number of cases that will require it is much fewer than when only oxygen therapy is used [11, 19]. Although more clinical trials are needed, there is agreement between the systematic reviews published in this regard that can encourage the generation of more evidence.

Our study has several strengths. Our search was exhaustive including gray literature and clinical trial registries. We follow the recommendations of the Cochrane collaboration and use GRADE to assess the quality of the evidence. Our principal limitation is the low number of patients included in the studies that does not allow us to conduct any subgroup or sensitivity analyses or the publication bias assessment. The quality of evidence in all outcomes, was judged as low quality, and was was related to the risk of bias and precision in the methods and low sample size.

In conclusion, we found, with low certainty, there was no significant difference between HFNC and CPAP in terms of risk of invasive mechanical ventilation. CPAP reduces de risk of therapeutic failure with a highest risk of non severe adverse events. More trials are needed to confirm theses results.

## Supplementary Information

**Additional file 1.**

## Data Availability

The data that support the findings of this study are available from the corresponding author upon reasonable request.
