# Supplementary material for: Systematic review and meta-analysis of efficacy and safety of continuous positive airways pressure versus high flow oxygen cannula in acute bronchiolitis
Source: BMC Pediatr. 2022 Dec 3;22:696. doi: 10.1186/s12887-022-03754-9 (PMC9719123; doi:10.1186/s12887-022-03754-9)
Supplement: Supplementary file 1 — Additional file 1. [file 12887_2022_3754_MOESM1_ESM.docx]

***Supplementary material***

**Full search strategy for all databases.**

| **Search electronic report #1** | |
| --- | --- |
| **Search type** | New |
| **Databases** | - Ovid MEDLINE(R) ALL <1946 to June 29, 2022> |
| **Platform** | Ovid.com |
| **Search date** | 30/06/2022 |
| **Update date** | Undefined |
| **Range of search date** | None |
| **Language restrictions** | None |
| **Other limits** | Cochrane Filter for RCT |
| **Search strategy (results)** | 1 exp bronchiolitis/ (9425)  2 bronchioliti$.tw. (12091)  3 1 or 2 (14532)  4 exp Continuous Positive Airway Pressure/ (8683)  5 (continuous adj5 positive adj5 pressure).tw. (11591)  6 (constant adj5 positive adj5 pressure).tw. (169)  7 (Airway adj5 Pressure adj5 Release adj5 Ventilation).tw. (307)  8 (Positive adj5 Airway adj5 Pressure).tw. (13286)  9 CPAP.tw. (9512)  10 CPPB.tw. (65)  11 CPPV.tw. (194)  12 APRV.tw. (189)  13 NCPAP.tw. (1170)  14 4 or 5 or 6 or 7 or 8 or 9 or 10 or 11 or 12 or 13 (19086)  15 exp Oxygen Inhalation Therapy/ (27882)  16 (Oxygen adj5 Therapy).tw. (15596)  17 (High adj5 Flow).tw. (24168)  18 High-Flow.tw. (9817)  19 HFNC.tw. (938)  20 HFNCT.tw. (1)  21 HFNO.tw. (115)  22 HHHFNC.tw. (59)  23 HHFNC.tw. (31)  24 15 or 16 or 17 or 18 or 19 or 20 or 21 or 22 or 23 (57775)  25 3 and 14 and 24 (93)  26 randomized controlled trial.pt. (571827)  27 controlled clinical trial.pt. (94924)  28 exp animals/ not exp humans/ (5022766)  29 26 or 27 (661878)  30 29 not 28 (647712)  31 25 and 30 (7) |
| **# of records identified** | 7 |
| **# of records after duplicates** | 7 |

| **Search electronic report #2** | |
| --- | --- |
| **Search type** | New |
| **Databases** | - Embase |
| **Platform** | Embase.com |
| **Search date** | 30/06/2022 |
| **Update date** | Undefined |
| **Range of search date** | None |
| **Language restrictions** | None |
| **Other limits** | Cochrane Filter for RCT |
| **Search strategy (results)** | #1. 'bronchiolitis'/exp 24,912  #2. bronchioliti*:ab,ti 18,631  #3. #1 OR #2 27,666  #4. 'continuous positive airway pressure'/exp 6,161  #5. (continuous NEAR/5 positive NEAR/5 16,902  pressure):ab,ti  #6. (constant NEAR/5 positive NEAR/5 pressure):ab,ti 257  #7. (airway NEAR/5 pressure NEAR/5 release NEAR/5 536  ventilation):ab,ti  #8. (positive NEAR/5 airway NEAR/5 pressure):ab,ti 20,063  #9. cpap:ab,ti 18,501  #10. cppb:ab,ti 94  #11. cppv:ab,ti 245  #12. aprv:ab,ti 437  #13. ncpap:ab,ti 1,798  #14. #4 OR #5 OR #6 OR #7 OR #8 OR #9 OR #10 OR #11 OR 32,472  #12 OR #13  #15. 'high flow nasal cannula therapy'/exp 3,699  #16. (oxygen NEAR/5 therapy):ab,ti 22,318  #17. (high NEAR/5 flow):ab,ti 33,457  #18. 'high flow':ab,ti 14,740  #19. hfnc:ab,ti 1,760  #20. hfnct:ab,ti 6  #21. hfno:ab,ti 275  #22. hhfnc:ab,ti 80  #23. hhhfnc:ab,ti 98  #24. #15 OR #16 OR #17 OR #18 OR #19 OR #20 OR #21 OR 55,629  #22 OR #23  #25. #3 AND #14 AND #24 186  #26. 'randomized controlled trial'/de 716,897  #27. 'controlled clinical trial'/de 437,242  #28. random*:ti,ab,tt 1,803,839  #29. 'randomization'/de 94,088  #30. 'intermethod comparison'/de 286,654  #31. placebo:ti,ab,tt 343,529  #32. compare:ti,tt OR compared:ti,tt OR 591,252  comparison:ti,tt  #33. (evaluated:ab OR evaluate:ab OR evaluating:ab OR 2,518,802  assessed:ab OR assess:ab) AND (compare:ab OR  compared:ab OR comparing:ab OR comparison:ab)  #34. (open NEXT/1 label):ti,ab,tt 97,380  #35. ((double OR single OR doubly OR singly) NEXT/1 259,750  (blind OR blinded OR blindly)):ti,ab,tt  #36. 'double blind procedure'/de 196,735  #37. (parallel NEXT/1 group*):ti,ab,tt 29,580  #38. crossover:ti,ab,tt OR 'cross over':ti,ab,tt 117,281  #39. ((assign* OR match OR matched OR allocation) 421,714  NEAR/6 (alternate OR group OR groups OR  intervention OR interventions OR patient OR  patients OR subject OR subjects OR participant OR  participants)):ti,ab,tt  #40. assigned:ti,ab,tt OR allocated:ti,ab,tt 450,613  #41. (controlled NEAR/8 (study OR design OR 419,568  trial)):ti,ab,tt  #42. volunteer:ti,ab,tt OR volunteers:ti,ab,tt 270,445  #43. 'human experiment'/de 583,062  #44. trial:ti,tt 368,215  #45. #26 OR #27 OR #28 OR #29 OR #30 OR #31 OR #32 OR 5,857,914  #33 OR #34 OR #35 OR #36 OR #37 OR #38 OR #39 OR  #40 OR #41 OR #42 OR #43 OR #44  #46. ((random* NEXT/1 sampl* NEAR/8 ('cross section*' 2,884  OR questionnaire* OR survey OR surveys OR  database OR databases)):ti,ab,tt) NOT  ('comparative study'/de OR 'controlled study'/de  OR 'randomised controlled':ti,ab,tt OR  'randomized controlled':ti,ab,tt OR 'randomly  assigned':ti,ab,tt)  #47. 'cross‐sectional study' NOT ('randomized 335,747  controlled trial'/de OR 'controlled clinical  trial'/de OR 'controlled study'/de OR 'randomised  controlled':ti,ab,tt OR 'randomized  controlled':ti,ab,tt OR 'control group':ti,ab,tt  OR 'control groups':ti,ab,tt)  #48. 'case control*':ti,ab,tt AND random*:ti,ab,tt NOT 19,876  ('randomised controlled':ti,ab,tt OR 'randomized  controlled':ti,ab,tt)  #49. 'systematic review':ti,tt NOT (trial:ti,tt OR 213,241  study:ti,tt)  #50. nonrandom*:ti,ab,tt NOT random*:ti,ab,tt 17,876  #51. 'random field*':ti,ab,tt 2,689  #52. ('random cluster' NEAR/4 sampl*):ti,ab,tt 1,563  #53. review:ab AND review:it NOT trial:ti,tt 992,890  #54. 'we searched':ab AND (review:ti,tt OR review:it) 42,320  #55. 'update review':ab 122  #56. (databases NEAR/5 searched):ab 55,296  #57. (rat:ti,tt OR rats:ti,tt OR mouse:ti,tt OR 1,163,362  mice:ti,tt OR swine:ti,tt OR porcine:ti,tt OR  murine:ti,tt OR sheep:ti,tt OR lambs:ti,tt OR  pigs:ti,tt OR piglets:ti,tt OR rabbit:ti,tt OR  rabbits:ti,tt OR cat:ti,tt OR cats:ti,tt OR  dog:ti,tt OR dogs:ti,tt OR cattle:ti,tt OR  bovine:ti,tt OR monkey:ti,tt OR monkeys:ti,tt OR  trout:ti,tt OR marmoset*:ti,tt) AND 'animal  experiment'/de  #58. 'animal experiment'/de NOT ('human experiment'/de 2,440,189  OR 'human'/de)  #59. #46 OR #47 OR #48 OR #49 OR #50 OR #51 OR #52 OR 4,016,973  #53 OR #54 OR #55 OR #56 OR #57 OR #58  #60. #45 NOT #59 5,188,288  #61. #25 AND #60 54 |
| **# of records identified** | 54 |
| **# of records after duplicates** | 54 |

| **Search electronic report #3** | |
| --- | --- |
| **Search type** | New |
| **Databases** | - EBM Reviews - Cochrane Central Register of Controlled Trials <May 2022> |
| **Platform** | Ovid.com |
| **Search date** | 30/06/2021 |
| **Update date** | Undefined |
| **Range of search date** | None |
| **Language restrictions** | None |
| **Other limits** | None |
| **Search strategy (results)** | 1 exp bronchiolitis/ (554)  2 bronchioliti$.tw. (1453)  3 1 or 2 (1502)  4 exp Continuous Positive Airway Pressure/ (1244)  5 (continuous adj5 positive adj5 pressure).tw. (4120)  6 (constant adj5 positive adj5 pressure).tw. (40)  7 (Airway adj5 Pressure adj5 Release adj5 Ventilation).tw. (89)  8 (Positive adj5 Airway adj5 Pressure).tw. (4786)  9 CPAP.tw. (4741)  10 CPPB.tw. (1)  11 CPPV.tw. (17)  12 APRV.tw. (78)  13 NCPAP.tw. (759)  14 4 or 5 or 6 or 7 or 8 or 9 or 10 or 11 or 12 or 13 (7059)  15 exp Oxygen Inhalation Therapy/ (1672)  16 (Oxygen adj5 Therapy).tw. (3644)  17 (High adj5 Flow).tw. (2778)  18 High-Flow.tw. (2219)  19 HFNC.tw. (596)  20 HFNCT.tw. (8)  21 HFNO.tw. (87)  22 HHHFNC.tw. (75)  23 HHFNC.tw. (26)  24 15 or 16 or 17 or 18 or 19 or 20 or 21 or 22 or 23 (6666)  25 3 and 14 and 24 (45) |
| **# of records identified** | 45 |
| **# of records after duplicates** | 42 |

| **Search electronic report #4** | |
| --- | --- |
| **Search type** | New |
| **Databases** | - CLINICALTRIALS.GOV |
| **Platform** | Ovid.com |
| **Search date** | 01/07/2022 |
| **Update date** | Undefined |
| **Range of search date** | None |
| **Language restrictions** | None |
| **Other limits** | NONE |
| **Search strategy (results)** | **cpap AND hfnc \| bronchiolitis** |
| **# of records identified** | 5 |

**Excluded articles and reasons**

- Only abstract

- Sinha R. Comparative study between CPAP and HHHFNC as respiratory support in moderate to severe Bronchiolitis in infants in PICU of a Tertiary care Hospital. Conference: PEDICON 2017. Avaliable in: <https://www.researchgate.net/publication/335756958_Comparative_study_between_CPAP_and_HHHNFheated_and_humidified_high_flow_nasal_cannula_as_respiratory_support_in_moderate_to_severe_bronchiolitis_in_infants_in_PICU_of_a_tertiary_care_hospital>
- Abboud P, Roth P, Yacoub N, Stolfi A. Efficacy of high flow/high humidity nasal cannula therapy in viral bronchiolitis. Critical Care Medicine [2015; 43(12):177](https://journals.lww.com/ccmjournal/toc/2015/12001) doi: 10.1097/01.ccm.0000474530.04830.28
- Mohanty S. High flow oxygen through nasal canula as alternative to continuous positive airway pressure ventilation as initial respiratory support for children with hypoxic respiratory failure. Pediatric Critical Care Medicine 2021; 22 - Issue Supplement 1 3S - p 1 doi: 10.1097/01.pcc.0000738112.96536.2b
- Included in following publication
- Milesi C, Essouri S, Pouyau R, Liet JM, Afanetti M, Baleine J, Durand S, Cambonie G, Breinig S, Javouhey E. Interest of high flow nasal cannula versus nasal continuous positive airway pressure during the management of severe bronchiolitis in infants: a multicenter randomized controlled trial. European journal of pediatrics 2016; 175(11): 1436
- Milesi C, Essouri S, Pouyau R, Liet JM, Afanetti M, Baleine J, Durand S, Durand P, Javouhey E, Roze JC, Dupont D, Cambonie G. Interest of high-flow nasal cannula (HFNC) versus nasal continuous positive airway pressure (nCPAP) during the initial management of severe bronchiolitis in infants: A multicenter randomized controlled trial. Annals of Intensive Care 2016; 6 (Suppl 1): O19
- Cesar R, Bispo B, Felix PH, Modolo MC, Cabo S, Souza A, Pizzini P, Horigoshi N, Rotta A. A randomized controlled trial of high-flow nasal cannula versus cpap in critical bronchiolitis. Critical Care Medicine 2018; 46(1): 553
- No intervention/comparation
- Milési C, Matecki S, Jaber S, Mura T, Jacquot A, Pidoux O, Chautemps N, Novais AR, Combes C, Picaud JC, Cambonie G. 6 cmH2O continuous positive airway pressure versus conventional oxygen therapy in severe viral bronchiolitis: a randomized trial. Pediatr Pulmonol. 2013 Jan;48(1):45-51. doi: 10.1002/ppul.22533.
- From clinical trials without results
- <https://clinicaltrials.gov/ct2/show/NCT01944995>
- <https://clinicaltrials.gov/ct2/show/NCT02457013>
- <https://clinicaltrials.gov/ct2/show/NCT04287960>
- <https://clinicaltrials.gov/show/NCT04650230>
